# The Effectiveness of Mostafa Maged Technique in Closure of the Episiotomy during Vaginal Delivery

**DOI:** 10.4314/ejhs.v33i1.7

**Published:** 2023-01

**Authors:** Mostafa Maged Ali

**Affiliations:** 1 Obstetric and Gynaecology Department, Egyptian Ministry of Health, Fayoum General Hospital, Fayoum, Egypt

**Keywords:** Mostafa Maged, Episiotomy, mediolateral, primigravida, vagina

## Abstract

**Background:**

This study is done to assess the effectiveness of Mostafa Maged technique in suturing the episiotomy.

**Methods:**

At the time of delivery, this technique will be applied to all women with episiotomy or perineal or vaginal tears. The technique employs absorbable vicryl threads with 75 mm round needles. Mostafa Maged technique includes the continuous suturing of the vaginal epithelium and the muscle layer. Evaluation of the perineal region within the next twenty-four hours prior to discharge searching for (edema-hematoma-septic wound - continence - ecchymosis - dyspareunia).

**Results:**

The current study included 50 patients. All patients had an episiotomy during delivery; 25 patients' episiotomies were sutured using Mostafa Maged technique, while the remaining patients' episiotomies were by regular traditional technique. Mostafa Maged technique has demonstrated efficacy in achieving adequate hemostasis and avoiding dead space formation during an episiotomy. It was found that 100 % of patients with Mostafa Maged technique have no dead space, and 95.8% of Mostafa Maged patients do not have vulval edema. The technique of Mostafa Maged has also proven effectiveness in achieving postoperative hemostasis. Unlike patients with regular maneuvers, 83.3% do not have dead space, and 83.3 % do not have vulval edema.

**Conclusions:**

Mostafa Maged technique is a simple technique and easy to apply when suturing episiotomy. Mostafa Maged technique is significantly superior to conventional maneuvers in preventing bleeding at the episiotomy site and preventing formation of dead space so achieving good hemostasis; therefore, it is highly recommended. I recommend more studies on efficacy of Mostafa Maged maneuver on large sample of patients.

## Introduction

An episiotomy is a surgical incision performed in the perineum to enable adequate space for delivering infants (1).

Three forms of episiotomy exist. Midline (median) episiotomy: a vertical incision done down from the center of the fourchette, encompassing underlying muscle, fascia, as well as vaginal mucosa. Mediolateral episiotomy is laterally directed at the same point, with the aim of avoiding the rectum. The mediolateral incision is roughly 45° from the centerline. Lateral episiotomy is nearly abandoned due to this region's excessive vascularity, which carries the risk of injuring Bartholin's duct and the bulbocavernosus muscle. Local tissue repair requires roughly seven days (2,3). A systematic review of the Cochrane database revealed that women with selective episiotomy reported 30 percent less severe perineal trauma at delivery compared to cases that underwent regular episiotomy. With respect to Apgar scores of less than seven at five minutes, the number of females getting a perineal infection, female numbers reporting painful sexual activity six months or more following childbirth, and urine incontinence six months or more following delivery, insignificant or no change was recorded. Nevertheless, other significant long-term outcomes as well as results were not documented in these investigations (fecal incontinence, rectal fistula, and urinary fistula). Consequently, it was determined that the reason for making regular episiotomies to avoid severe perineal injuries was unwarranted and that routine episiotomy provided no advantages to the mother or the infant (4).

Although the episiotomy technique is taken into account, mediolateral episiotomy does not seem to be protective against sonographically or clinically diagnosed obstetrical anal sphincter injuries (OASIS), which are linked to diminished orgasm, arousal, sexual desire, as well as sexual functioning in the first five years following childbirth (5).

Episiotomy's prophylactic use in critical cases, e.g., non-reassuring fetal cardiac patterns, occiputposterior position, shoulder dystocia, fetal macrosomia, and instrumental deliveries, does not prevent perineal tears of the third or fourth degree (6).

Even though multiple perineal repair techniques have been documented, obstetricians agree that the optimal repair technique is what results in less pain in the long and short term, consumes less time to perform and less material use, requires fewer sutures, permits intercourse sooner resumption, and a decreased resuturing frequency (7).

## Methods

This clinical trial study was conducted to 50 pregnant women, aged 18 years or more and who are suitable for normal vaginal delivery, they were enrolled to this study after taking an ethical committee approval in Egypt. A detailed history was taken from all participants, and general examination to exclude the presence of any disorders. Obstetric examinations were performed. Verbal and written consents from all patients are obtained to be recruited in this study.

All relevant information, like the purpose and methodology of the experiment, was explained to study participants beforehand, and informed consent was obtained. All procedures of the present study were conducted in compliance with the Helsinki declaration for research on human beings. The study was approved by the research ethics committee.

Clinical trial number (ClinicalTrials.gov Identifier) is NCT05247073

**Active Comparator of this study (Procedure of Mostafa Maged technique**: Patients of study group with Mostafa Maged technique for closure of the episiotomy. The vagina will be stitched with the Mostafa Maged technique, The Mostafa Maged four-stitch technique uses absorbable vicryl threads with round needles 75 mm. The technique will prevent dead space formation, Good and tight hemostasis of the episiotomy strong approximation of the two edges of the episiotomy.

**Procedure of Mostafa Maged technique**:

1. Mostafa Maged four-stitch technique for closure of the episiotomy.
2. Identification of the apex of the episiotomy, then a simple suture is taken (0.5 cm) behind the apex of the episiotomy. First, the needle is inserted at the vaginal mucosa (epithelium) of the right edge of the episiotomy then extracts the needle.
3. The second stitch is inserted on the muscle layer of the same side (right side) of the episiotomy cutting edge then extracting the needle.
4. Then, insert the needle again on the left side of the episiotomy incision in the muscle layer on the left side of the episiotomy incision directing the tip of the needle upwards parallel to the second stitch taken.
5. The fourth step is inserting the needle in the vaginal mucosa (epithelium) of the left side parallel to the first stitch. Continue suturing the episiotomy incision continuously in the same way till reaching the remnant of the hymen (fourchette). Then, I make a loop knot at the fourchette. Then, suturing the superficial perineal muscle in a continuous manner and the skin in a subcuticular manner as well. Mostafa Maged technique is illustrated in a model of uterus in Figure (2).

**Figure:**
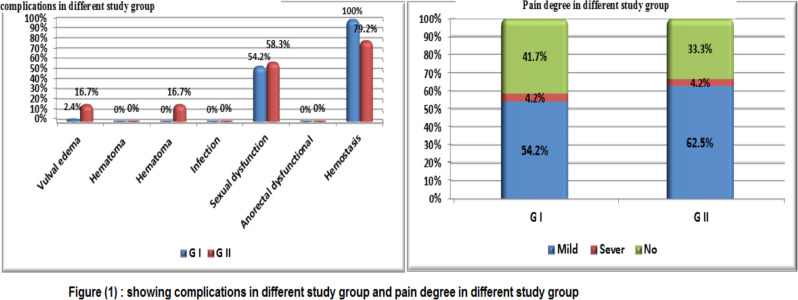


There is a case series of this (Mostafa Maged technique) recently published to reveal this technique (13).

**Patients of controlled group**: The vagina will be stitched using a continuous locking stitch and the perineal muscles and skin are repaired using approximately three or four individual stitches, each needing to be knotted separately to prevent them from dislodging.

Procedure (Patients with routine traditional closure of the episiotomy): patients of controlled group with routine closure of episiotomy

Perineal trauma is traditionally repaired in three stages: a continuous locking stitch is inserted to close the vaginal trauma, commencing at the apex of the wound and finishing at the level of the fourchette with a loop knot. The perineal muscles are then re-approximated with three or four interrupted sutures and finally, the perineal skin is closed by inserting continuous subcutaneous or interrupted transcutaneous stitches.

The skin is then closed with inverted interrupted stitches placed in the subcutaneous tissue a few millimeters under the perineal skin edges (not trans-cutaneously).

While primigravida patients having episiotomies or tears in the vagina and age between 18 to 40 years old were included in the study; whereas, smokers, diabetics, morbidly obese patients, cases with chronic diseases such as renal diseases and cases with 3^rd^ and 4^th^ perineal tears were excluded.

**Primary Outcome Measures:**

Heamostasis of the episiotomy [Time Frame: 4 weeks after delivery] (Bleeding from the epistiomy or heamatoma at the epistomy).

No edema at the site of episiotomy [Time Frame: 4 weeks after delivery] (Swelling or ecchymosis and edema at the edges of episiotomy).

No infection at the episiotomy [Time Frame: 4 weeks after delivery] (Redness, hotness and bad odour of vaginal discharge)

**Secondary Outcome Measures:**

Sexual dysfunction (pain during sexual intercourse) [Time Frame: 4 weeks after delivery] (Pain during sexual intercourse)

Anorectal dysfunction [Time Frame: 4 weeks after delivery] (Inability to control passage of stool or flatus or both).

**Statistical analysis**: Data collection and coding were performed to enable data manipulation as well as double entered into Microsoft Access, and the analysis was done utilizing version 22 of the Statistical Package of Social Science (SPSS) (SPSS Inc., Chicago, IL, USA). In addition, a simple descriptive analysis was performed using percentages and numbers of qualitative data, standard deviations for the dispersion of quantitative parametric data, as well as arithmetic means as central tendency measurement.

**For quantitative data**: Independent samples t-test was utilized for comparing quantitative measures between two independent groups.

**For qualitative data**: The Chi-square test was utilized for comparing two of more than two qualitative groups. The p-value< 0.05 was considered statistically significant.

## Results

Table 1 illustrates that there was no statistical significance with p-value >0.05 as regards age, weight, height, BMI and blood pressure, between both stud groups.

**Table 1:** Comparisons of age, anthropometric measurements, and vital signs in different study groups.

Variables	Mostafa Maged technique (N=25)	Regular maneuver (N=25)	P-value
		
	Mean ± SD	Mean ± SD	
Age (years)	24.8±7.5	24.5±5.1	0.86
Weight (kg)	73.4±4.4	75.3±4.8	0.06
Height (cm)	1.68±0.02	1.69±0.03	0.3
BMI (kg/m2)	25.6±2.03	26.3±2.02	0.22
Systolic BP	108±15.8	109.6±13.1	0.69
Diastolic BP	75±8.2	73.8±8.8	0.62

*significance difference p-value <0.05

The table (2) illustrated that there was no statistical significance with p-value >0.05 as regards gestational age, parity and number of fetus between both stud groups.

**Table (2):** Comparisons of obstetric data in different study groups

Variables	Mostafa Maged technique (N=25)		Regular maneuver (N=25)		P-value
		
	Mean ± SD		Mean ± SD		
Gestational age (wk)	37.2±1.7		37.2±1.6		0.93
**Parity**					
Primi	25	100%	25	100%	1
Multi	0	0%	0	0%	
**Number of fetus**					
Single	25	100%	25	100%	1
Twins	0	0%	0	0%	

*significance difference p-value <0.05

The table (3) illustrated that there was a statistical significant with p-value <0.05 as regards hemostasis complication between both stud groups with higher percentage among group of Mostafa Maged technique.

**Table 3:** Comparisons of episiotomy complications in different study groups

Variables	Mostafa Maged technique (N=24)		Regular maneuver (N=24)		P-value
		
	No. (%)		No. (%)		
**Vulval edema**					
Yes	1	4.2%	4	16.7%	0.34
No	23	95.8%	20	83.3%	
**Hematoma**					
Yes	0	0%	0	0%	1
No	24	100%	24	100%	
**Dead space**					
Yes	0	0%	4	16.7%	0.10
No	24	100%	20	83.3%	
**Infection**					
Yes	0	0%	0	0%	1
No	24	100%	24	100%	
**Sexual dysfunction**					
Yes	13	54.2%	14	58.3%	0.9
No	11	45.8%	10	41.7%	
**Ano-rectal dysfunction**					
Yes	0	0%	0	0%	1
No	24	100%	24	100%	
**Pain at site**					
Mild	13	54.2%	15	62.5%	0.83
Sever	1	4.2%	1	4.2%	
No	10	41.7%	8	33.3%	
**Hemostasis**					
Yes	24	100%	19	79.2%	**0.05** *
No	0	0%	5	20.8%	

* significance difference p-value <0.05

On the other hands, there was no statistical significant with p-value >0.05 as regards different complication as (vulver edema, hematoma, dead space, infection, sexual dysfunction, ano-rectal dysfunction, and pain) between both stud groups. In figure (1) we see the summary of the statistical analysis of this study. The view of episiotomy line after being closed by Mostafa Maged maneuver has been shown in figures 2.

**Figure:**
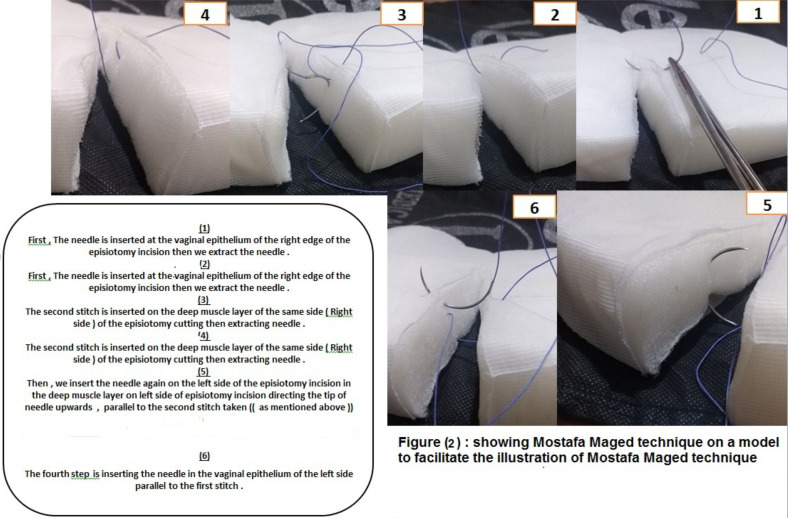


## Discussion

This study aims to compare the effectiveness of Mostafa Maged maneuver in avoiding complications that can arise during episiotomy closure. This study included fifty divided into two groups: 25 patients were informed of Mostafa Maged episiotomy closure procedure, and the remaining 25 patients were informed of the standard episiotomy closure procedure. Therefore, we have utilized the mediolateral episiotomy incision in the current study for both groups to avoid the occurrence of fecal incontinence.

For the future, we would suggest more studies about the effectiveness of Mostafa Maged maneuver on a large sample of patients. Until the results of these future clinical trials become available, we suggest performing Mostafa Maged maneuver during the episiotomy. Mostafa Maged technique is an easy procedure that can be easily applied while suturing the episiotomy. Mostafa Maged technique is significantly better than traditional maneuvers in preventing vulval edema and blocking dead space formation; consequently, it is highly recommended.

Regarding perineal pain, it has been established for over seventy years that continuous repair methods for episiotomy are superior to interrupted suture techniques. Fleming discovered that continuous suturing methods with subcuticular sutures of perineal skin were associated with a lower level of perineal pain than the traditional interrupted suturing method (8,9).

Regarding the time of full recovery, there was a delay in those with wound infection or deep perineal tears, which was more common with median episiotomy than with mediolateral episiotomy, consistent with the findings of Owen and Hauth and Aytan *et al* (10).

Consequently, we have performed a mediolateral incision in our study. If the episiotomy repair does not entirely halt the bleeding, a hematoma may develop beyond the suture line. The most prevalent varieties of hematoma include vulvovaginal and vaginal hematomas, as well as uncommon paravaginal hematomas, which may be very tiny, but also huge retroperitoneal hematomas, which are potentially lethal. The most common symptoms are rectal pressure and discomfort and a fluctuating palpable lump in the iliac area. When a hematoma forms, the incision must be repeated in order to drain the blood accumulation. In situations of lesser hematomas, the episiotomy must be sutured again, or the location may be left to heal spontaneously over time (11).

Therefore, we must confirm that Mostafa Maged technique has demonstrated efficacy in achieving the hemostasis effect after the closure of the episiotomy. As evidenced by the results of this study, no hematomas are formed with Mostafa Maged technique when compared to regular maneuvers to close episiotomies.

In addition, the results of the current study demonstrate the efficacy of Mostafa Maged technique in leaving no dead space (zero percent) after episiotomy closure and its extraordinary hemostatic effect in that both edges of the episiotomy are firmly attached (100%), unlike the regular maneuver (79.2 %).

Signorello *et al* reported that females with midline episiotomies are more prone to develop fecal incontinence at three as well as six months postpartum than females with intact perineum (12, 13).
